# Evaluating Cost-Effective Methods for Rapid and Repeatable National Scale Detection and Mapping of Invasive Species Spread

**DOI:** 10.1038/s41598-019-43729-y

**Published:** 2019-05-10

**Authors:** Ruth A. Aschim, Ryan K. Brook

**Affiliations:** Department of Animal and Poultry Science, College of Agriculture and Bioresources, University of Saskatchewan, 51 Campus Drive, Saskatoon, SK S7N 5A8 Canada

**Keywords:** Conservation biology, Invasive species

## Abstract

Invasive species can spread rapidly at local and national scales, creating significant environmental and economic impacts. A central problem in mitigation efforts is identifying methods that can rapidly detect invasive species in a cost-effective and repeatable manner. This challenge is particularly acute for species that can spread over large areas (>1 million km^2^). Wild pigs (*Sus scrofa*) are one of the most prolific invasive mammals on Earth and cause extensive damage to agricultural crops, native ecosystems, and livestock, and are reservoirs of disease. They have spread from their native range in Eurasia and North Africa into large areas of Australia, Africa, South America, and North America. We show that the range of invasive wild pigs has increased exponentially in Canada over the last 27 years following initial and ongoing releases and escapes from domestic wild boar farms. The cumulative range of wild pigs across Canada is 777,783 km^2^, with the majority of wild pig distribution occurring in the Prairie Provinces. We evaluate eight different data collection and evaluation/validation methods for mapping invasive species over large areas, and assess their benefits and limitations. Our findings effectively map the spread of a highly invasive large mammal and demonstrate that management efforts should ideally rely on a set of complementary independent monitoring methods. Mapping and evaluating resulting species occurrences provide baseline maps against which future changes can be rapidly evaluated.

## Introduction

The Anthropocene is the current geological age on earth, characterized by the dominant influence of humans on the environment, including impacts on native ecosystems and spread of invasive species globally^1^. Indeed, invasive species have been identified as one of the greatest threats to global biodiversity^2^. Human activities can facilitate the spread of invasive species, directly through intentional and unintentional movements of plants and animals, and indirectly through habitat fragmentation and change associated with agriculture, urban expansion, and other anthropogenic land-use changes, climate change, and over-harvest of native species^3–5^. Efforts to mitigate the spread of invasive species have been limited by the lack of timely and accurate maps of occurrences and spatial expansion, especially over very large areas (>1 million km^2^). Processes required to complete comprehensive national scale mapping of a species are limited by project budgets. New approaches are needed that are cost-effective and repeatable, especially for species expected to expand rapidly over large areas and that are associated with greatest impact.

Wild pigs (*Sus scrofa*), also referred to as feral swine, wild hogs, or feral hogs^6^, currently have the largest global range of any non-domesticated terrestrial mammal on earth^7^. Native to Eurasia and part of North Africa, wild pigs have expanded their range, primarily through human introductions combined with natural dispersal, across all continents except Antarctica^7,8^. The broad geographic extent of their native range, coupled with the generalist nature of the species has allowed wild pigs to easily adapt and survive in new environments that span a broad range of climate, habitat, and resources^9,10^. The widespread success of wild pigs is explained by their extremely high fecundity^11^, early sexual maturity^12^, plastic diet^7^, long lifespans^13^, and highly adaptive nature^9^.

Wild pigs are an invasive species in North America and are descendants of Eurasian wild boar (*S. scrofa scrofa*), domestic pigs (*S. scrofa domesticus*), and hybrids of the two^14,15^. Long-established populations have existed in southern parts of the U.S. for hundreds of years, with areas of high population densities in Texas, Florida, and California. In the continental United States, there has been a well-documented expansion in the distribution and abundance of wild pigs in recent decades, from 17 to 38 states during the last 30 years^16^. Wild pigs were first introduced to Canada during a federal and provincial agriculture diversification initiative in the 1980’s and 1990’s to diversify livestock species and supplement producer incomes^17,18^. Escapes and intentional releases from domestic wild boar farms have led to the feral populations that are established on the Canadian Prairies^18,19^. Brook and van Beest^18^ provided a coarse-scale distribution of wild pigs in Saskatchewan at the Rural Municipality level. However, prior to this current study there has not been a comprehensive national scale map of the species range in Canada.

Wild pigs are considered to be the most damaging invasive species in the U.S.^20^, posing numerous ecological and socio-economic threats within their introduced range. Referred to as ecological train wrecks, wild pigs alter ecosystem processes, vegetation successional stages, nutrient cycles, and cause erosion, sedimentation, and eutrophication to riparian areas and water bodies^7,16,20–23^. The generalist nature and plastic diet of wild pigs allows them to utilize and compete for a wide variety of resources, as well as predate small mammals, amphibians, invertebrates, and ground nests^16^. The significant disturbance of habitat, resources, and ecosystem processes has direct and indirect effects on native wildlife and has the ability to decrease biodiversity and cause extirpations and extinctions^20,24,25^. Species extirpations and population declines as a result of wild pig presence have been documented in the United States, Galapagos Islands, and Australia^16,26–28^. In some areas within wild pig’s introduced range, populations have expanded to the point where eradication is no longer feasible^29^. Negative impacts associated with wild pigs have been well-documented across Europe, Australia, and the United States; however, these negative impacts have not been characterized in Canada.

A key challenge in managing rapidly expanding invasive species such as wild pigs at national and continental scales is having up-to-date information on their spatial distribution. Mapping the locations of invasive species is central to guiding effective management and is essential to determine if control efforts are effective at controlling and limiting, or even reducing, their spatial expansion^30^. However, identifying cost-effective methods to accurately and repeatedly map a species at a national scale represents a significant time and financial commitment. Conventional ecological monitoring used for large mammals such as aerial surveys, trail cameras, and mark-recapture can be effective at relatively small scales (<100,000km^2^), but become time and cost prohibitive at much larger scales. The use of local and traditional knowledge accumulated by people living and working on the land through personal observations and shared knowledge has been used for documenting species occurrences^31,32^. Such data can be collected using personal interviews, mail surveys, internet surveys, telephone surveys, and open source mapping^33,34^. Similarly, citizen science engages large numbers of lay people in collecting species occurrence data, such as the annual Breeding Bird Count and the Christmas Bird Count across the United States and Canada^35^, though this also requires considerable logistics coordination and has rarely been used for large mammals. Efforts to incorporate local knowledge in data collection using rigorous social science methods have rarely been applied over large areas (>1 million km^2^). Much work remains to evaluate the efficacy of these methods and determine the benefits and limitations of the different approaches in the face of immediate needs for detailed information on invasive species in general and wild pigs in Canada specifically. As such, the objectives of this study were to: (1) identify the past and current spatial distribution of wild pigs across Canada, and (2) evaluate the benefits and limitations of four different data collection methods and four different evaluation/validation approaches for national-scale, repeatable mapping of wild pig spatial distribution.

## Results

**Figure 1:**
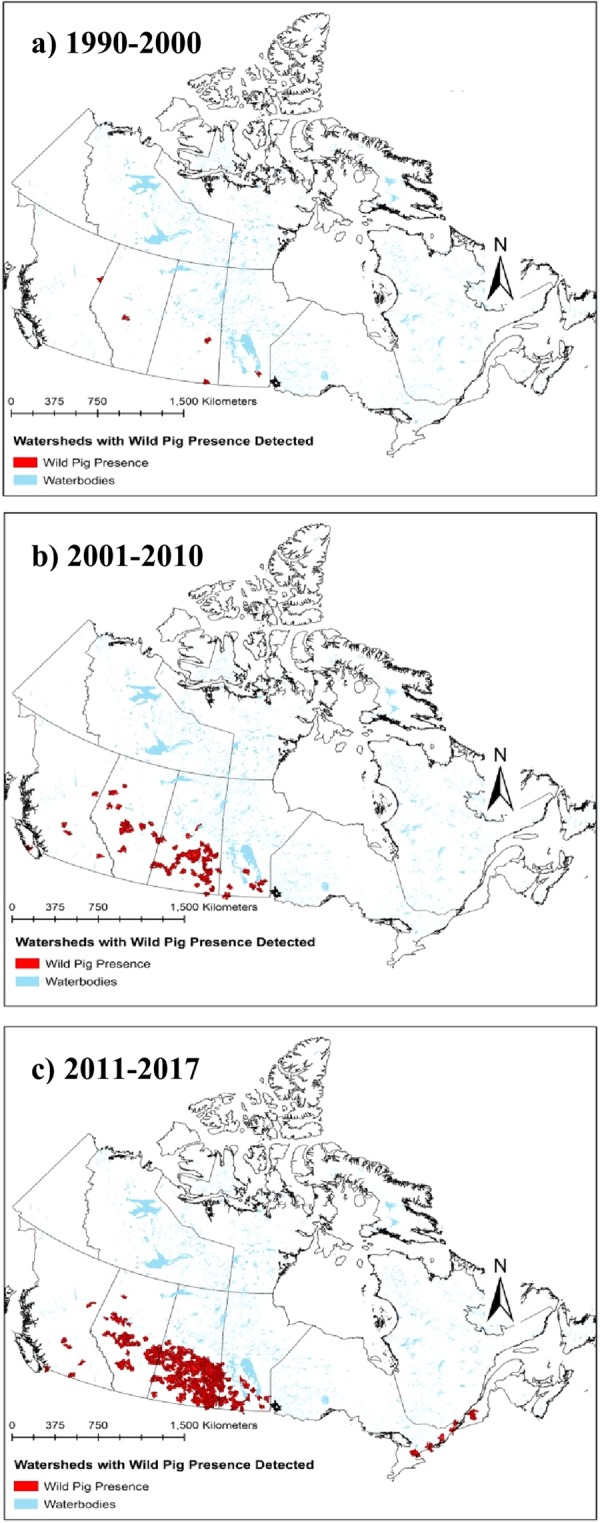
Spatial expansion of wild pigs in Canada from the period of initial escapes and releases (1990) to present, based on combined results from point occurrences obtained from national scale expert interviews, stakeholder snowball sampling, rural telephone survey, and bounty data. 939 wild pig point occurrences were obtained from all methods. Watersheds where wild pigs were detected are mapped for three time periods, (**a**) 1990–2000, (**b**) 2001–2010, and (**c**) 2011–2017.

**Figure 2:**
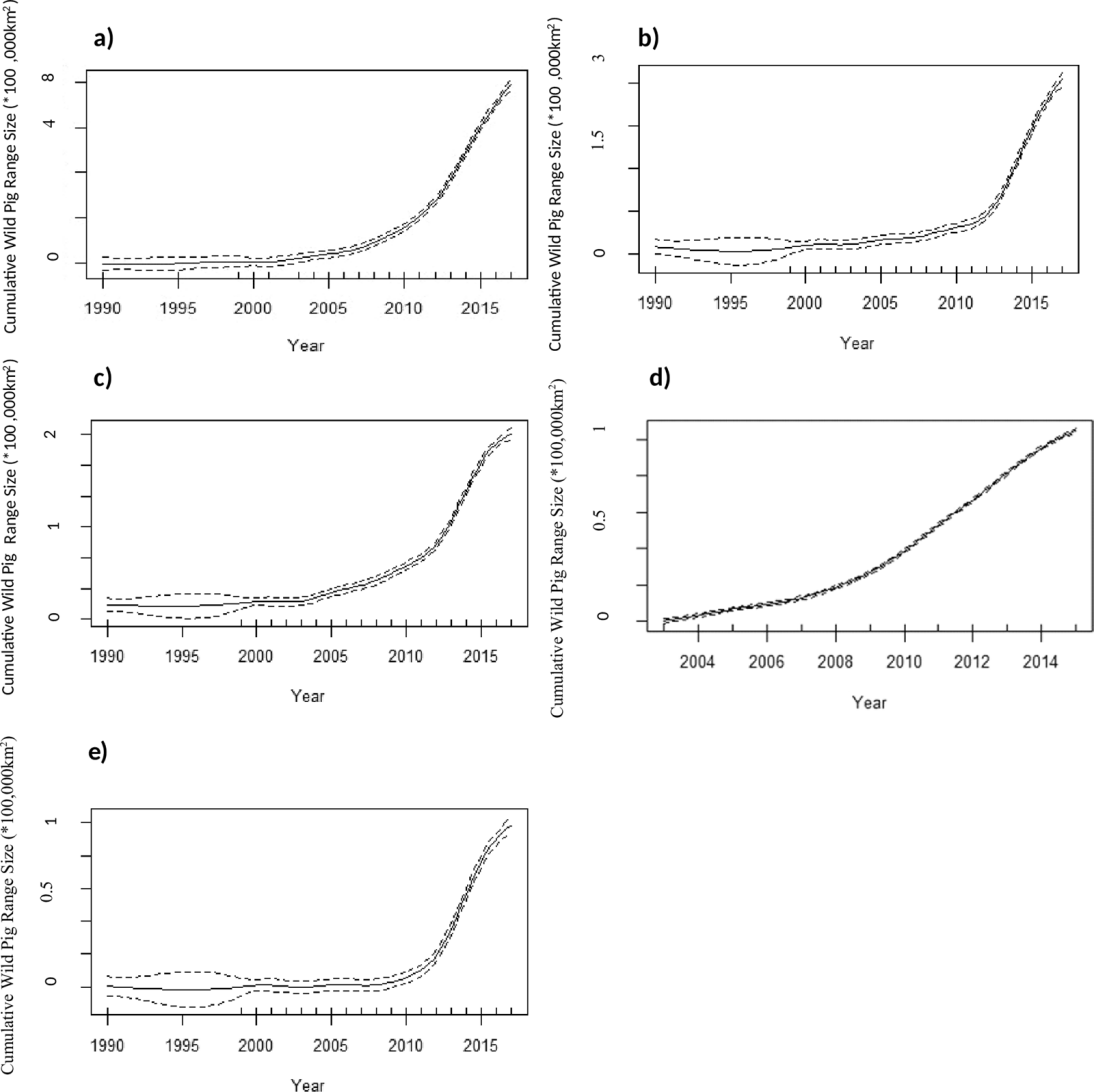
(**a**) Cumulative range size of wild pigs in Canada mapped by occupied watersheds per year from 1990–2017 using (**a**) combined results from point occurrences obtained from all methods; (**b**) stakeholder snowball sampling; (**c**) national scale expert interviews; (**d**) bounty data; and (**e**) national scale rural telephone survey. Results were modelled using a generalized additive model in R (n = 19, R^2^ = 0.998, p <0.001) along with a 95% confidence interval.

**Figure 3:**
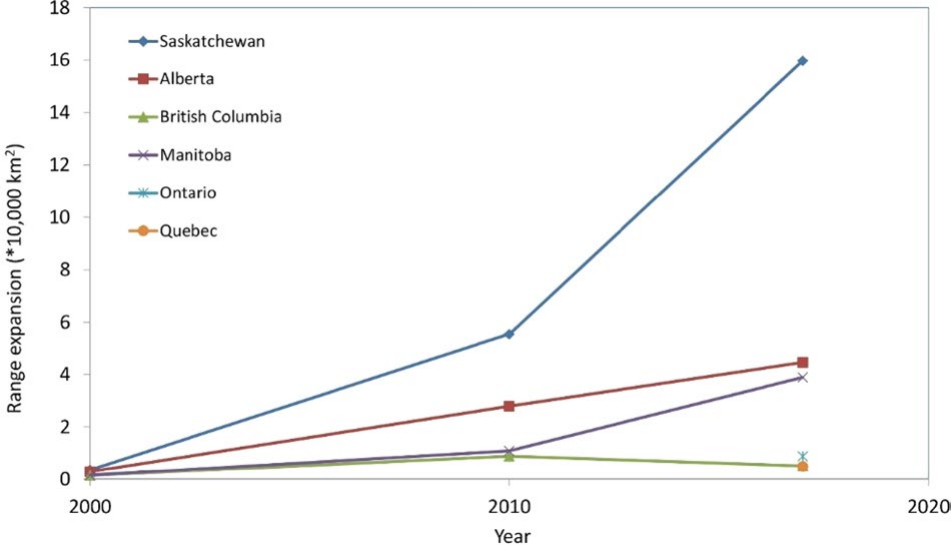
Increase per decade in range size of wild pigs in each of the seven Canadian provinces confirmed to have wild pigs. Range mapped by occupied watersheds (1990–2017) using combined results from point occurrences obtained from national scale expert interviews, stakeholder snowball sampling, a rural telephone survey, and bounty data.

Wild pig occurrence data were collected from four independent data collection methods (stakeholder snowball sampling, expert interviews, bounty data, and rural telephone survey) and were used directly to develop maps of wild pig distribution across Canada using all datasets combined for three time periods, 1990–2000, 2001–2010, and 2011–2017 (Fig. 1). An additional four methods (GPS collars, citizen science photos, research trail camera networks, and media search) were used to evaluate benefits and limitations of methods and validate observations. A total of 1,489 occupied watersheds were identified, out of 37,578 watersheds in the study area (3.9% occupied by wild pigs), based on pooled results of all data collection methods across all years. The area of watersheds occupied in Canada has increased exponentially from 1990 to 2017 (Fig. 2). The large majority of the spatial expansion (92%) occurred in the three prairie provinces of Alberta, Saskatchewan, and Manitoba (Fig. 3). Indeed 58% of the national spread of wild pigs occurred within Saskatchewan. Wild pigs are also established in localized populations in British Columbia, Ontario, and Quebec. Of the ten provinces in Canada, only the four eastern provinces in Atlantic Canada (Newfoundland and Labrador, New Brunswick, Prince Edward Island, and Nova Scotia; 5% of Canada combined) have no confirmed sightings of wild pigs.

Based on analysis at the Level 9 watershed, the cumulative range of wild pigs in Canada is 777,783 km^2^. The average annual cumulative increase in wild pig range from the period 1990–2017 was 40,936 km^2^. The greatest increase in range expansion has occurred in the current time period (2011–2017), with an average annual cumulative increase of 88,094 km^2^.

As expected, the number of detected watersheds in Canada occupied by wild pigs was different among sampling methods (Table 1). There was an overall high consistency in the spatial distribution of occupied watersheds for each method. However, specific correspondence of individual occupied watersheds between data collection methods was low overall (<50%) for all methods (Table 2). Correspondence was strongest between the occupied watersheds determined using expert interviews and stakeholder snowball sampling methods (21% correspondence). The number of occupied watersheds was significantly and positively associated with the level of correspondence among all possible pairs of data collection methods (R^2^ = 0.90, df = 5, p < 0.001).

**Table 1:** Comparison of the number and distribution of watersheds in Canada with wild pig occurrences, based on four unique monitoring strategies.

Data Collection Method	Years Covered in Data Collection	Total Number of Watersheds Detected	Number of Unique Watersheds Detected^a^
Stakeholder	1990–2017	639	297
Expert	1990–2017	522	177
Phone	1990–2016	215	25
Bounty	2003–2016	113	32
**Total**	—	**1,489**	**531**

^a^Watershed detected by the specified method only.

**Table 2:** Number of duplicate watersheds with wild pig presence detected and the associated correspondence between each pair of the four data collection and mapping methods used to identify wild pig distribution across Canada (1990–2017).

Data Collection Pairs	Duplicate Watersheds With Wild Pigs Detected^a^	% Correspondence
Stakeholder-Expert	240	21
Stakeholder-Phone	89	10
Stakeholder-Bounty^b^	13	2
Expert-Phone	69	9
Expert-Bounty^b^	36	6
Phone-Bounty^b^	32	10
Stakeholder-ALL	342	54
Expert-ALL	345	66
Phone-ALL	190	88
Bounty-ALL	81	72

^a^Occupied watersheds that were detected by both methods.

^b^Only Alberta locations were used for correspondence with bounty as this is the only province that implemented a bounty during this study.

**Figure 4:**
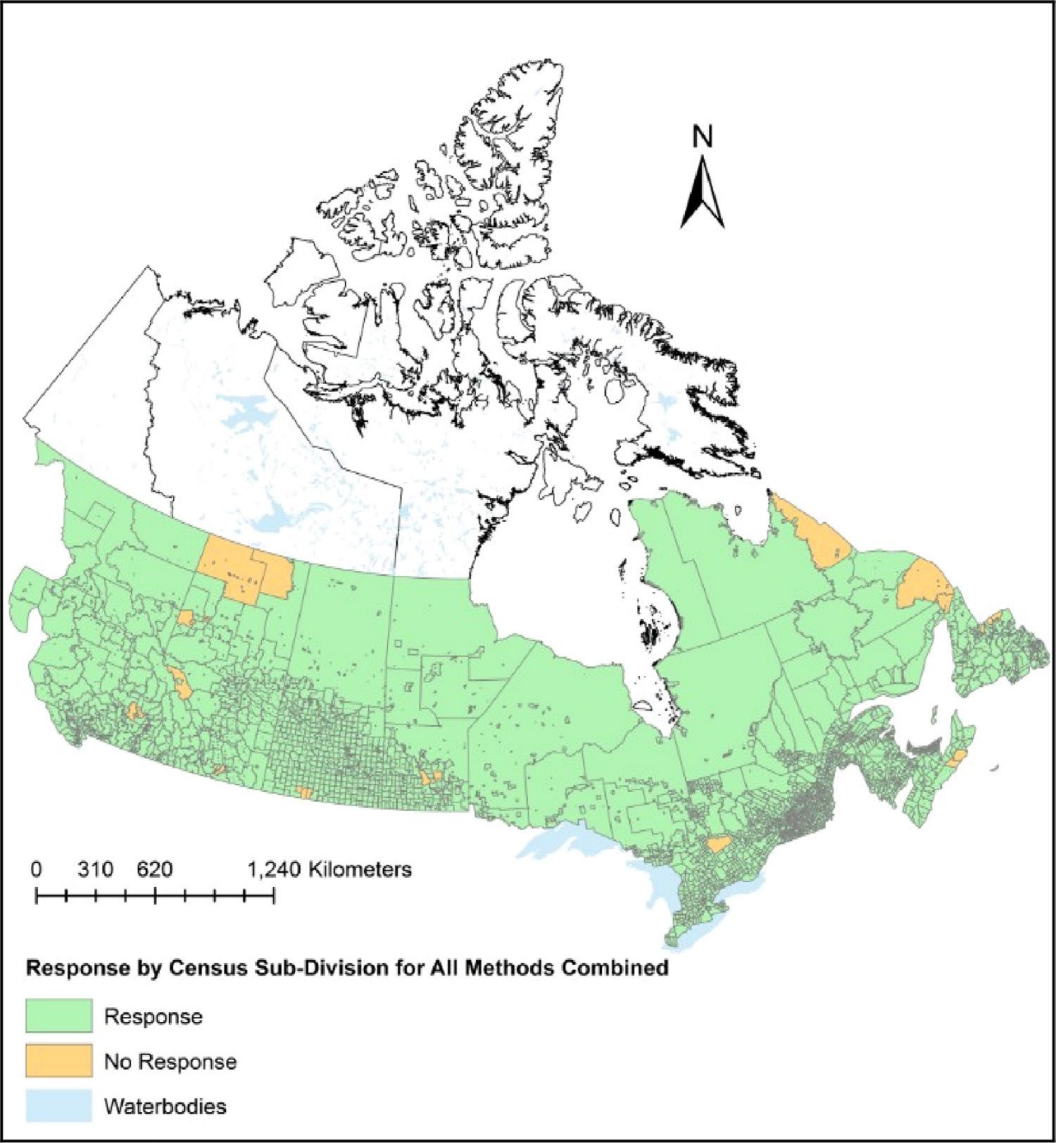
Response from expert interviews, stakeholder snowball sampling, rural telephone survey, and bounty data regarding wild pig inquiries collected from 2014–2017. Stratified across the study area at the Canadian census sub-division level (average area 1,195 km^2^).

Overall mean response rate for the expert interviews was 51% (S.E. = 5.9). The mean number of completed districts within provinces was 55% (S.E. = 7.7). Total number of individual participants in the stakeholder snowball sampling method and rural telephone survey was 275 and 3,000 respectively. 272 detections were received from the bounty data. Response from all methods combined provided 95.5% coverage of the study area using the Canadian census sub-division as stratification units (Fig. 4).

Each of the eight research methods assessed to identify and evaluate wild pig distribution had important benefits and limitations and were viewed by participants with varying degrees of creditability and spatial accuracy (Table 3). The three social methods (expert interviews, stakeholder snowball sampling, and rural telephone survey) resulted in the majority (94%) of all watersheds detected with wild pigs in this study, with stakeholder snowball sampling providing the greatest number of occupied watersheds. Total cost of the data collection and mapping methods during the study period was $125,621; with an average of $294 per wild pig occurrence (range $50–$852). Cost of evaluation and validation methods totaled $671,000; with an average of $247 per wild pig occurrence (range $4–$644). Overall cost for all eight methods used was $796,621.

**Table 3:** Summary of financial costs, benefits, and limitations of (a) four different data collection methods for detecting invasive wild pig occurrences and occupied watersheds across Canada collected during 2014–2017 that cover the time period 1990–2017 and (b) four evaluation methods.

Method^a^	Key Benefits^c^	Key Limitations^c^	Perceived Credibility^c^	Spatial Error^d^ (m)	Years	Total Number of Wild Pig Occurrences Detected	Total Number of Watersheds Detected^e^	Project Cost^f^	$/Wild Pig Occurrence	$/Occupied Watershed
**Detection and Mapping**										
Expert Elicitation*	targeted, systematic coverage	not all are ‘experts’	moderate	400	1990–2017	203	522	$41,500	$204	$80
Stakeholder Snowball Sampling*	highly targeted	not systematic, potential biases	moderate	400	1990–2017	373	639	$25,300	$68	$40
Rural Telephone Survey*	representative sample	non-targeted, small number of questions to ask	moderate	400	1990–2016	53	215	$45,171	$852	$210
Bounty Data*^b^	large scale coverage, one province only	some potential for misreporting of locations	moderate	400	2003–2016	272	113	$13,650^g^	$50^b^	$121^b^
**COMBINED**	**Maximizes benefits**	**Minimizes limitations**	**Moderate**	$$\bar{{\bf{x}}}$$ **= 400**	**1990–2017**	**∑ = 901**	**∑ = 1,489**	**∑ = $125,621**	$$\bar{{\bf{x}}}$$ **= $294**	$$\bar{{\bf{x}}}$$ **= $113**
**Evaluation and Validation**										
Research Trail Camera Networks	high spatial accuracy, unbiased	limited scope, high total cost, cameras stolen	high	15	2011–2013	45	6	$29,000	$644	$4,833
Citizen Science Photos	high spatial accuracy	not systematic	moderate	50	1990–2017	508	9	$2,000	$4	$222
Media Search	national coverage, easily searchable	many occurrences go unreported	moderate	1500+	1990–2017	3	1	$1,000	$333	$1,000
GPS Collars	high spatial accuracy, unbiased	relatively low spatial coverage	high	15	2015–2017	95,400	14	$639,000	$7	$45,643
**COMBINED**	**High spatial accuracy**	**Fine-scale, biased**	**Moderate**	$$\bar{{\bf{x}}}$$ **= 395**	**1990–2017**	**∑ = 95,956**	**∑ = 30**	**∑ = $671,000**	$$\bar{{\bf{x}}}$$ **= $247**	$$\bar{{\bf{x}}}$$ **= $12,925**
**ALL COMBINED**	**Maximizes benefits**	**Minimizes limitations**	**Very high**	$$\bar{{\bf{x}}}$$ **= 398**	**1990–2017**	**∑ = 96,857**	**∑ = 1,519**	**∑ = $796,621**	$$\bar{{\bf{x}}}$$ **= $271**	$$\bar{{\bf{x}}}$$ **= $6,519**

^a^Methods marked with *were used for national scale mapping, other methods were used to evaluate detection methods and validate mapping. As such, total number of watersheds detected in Table 1 and Table 3 are different.

^b^The bounty program was only run in the province of Alberta.

^c^As estimated by the researchers based on experience during this project.

^d^Estimated based on map scales used (social data from interviews/surveys) or field measures of spatial error using GPS (ecological data including trail cameras and GPS collars).

^e^Total number of watersheds detected with occurrences of wild pigs including any repeated detections.

^f^Project costs include cost of travel, contract phone surveys, all equipment and student and research assistant time. Principle Investigator salary was included for project management that was provided as in-kind from the University of Saskatchewan.

^g^Includes $50 paid for each set of wild pig ears turned in. Other program administration costs were not available but will increase total program costs.

## Discussion

Invasive wild pigs are widespread and rapidly expanding their range in Canada, creating significant risks to the sustainability of native ecosystems and agricultural and livestock production following their escapes and purposeful releases from multiple sources at wild boar meat and penned shoot farms, starting in 1990. This study has produced the first published maps that document the spread of wild pigs across Canada. We show that the majority of the expansion has occurred in the three prairie provinces of Alberta, Manitoba, and especially Saskatchewan. Introduction events and the abundance of agriculture as a high-quality food source exert the greatest effect on the success of wild pig establishment and growth^36,37^. The Prairie Provinces historically and currently contain the highest number of wild boar farms in Canada^19^, and are dominated by an agricultural landscape, both of which are expected to have influenced the establishment success and rapid spread of wild pigs. We expect that given the range of available food resources and habitats^18^, high reproduction rates with an average of 6 young per female^38^, and the overall absence of national and provincial management plans and control efforts, wild pig populations and range will continue to expand exponentially over the next decade at least. Our results are consistent with the rapid expansion of wild pig populations in the United States^16,39^ and most other areas of their native and introduced range^40–42^. Many areas in Canada are susceptible to wild pig expansion, especially those that are comprised of ample, energy-rich food resources from agriculture crops^10^, forest cover^37^, relatively low predator densities^37^, and repeated introduction/re-introduction events^19^.

Our finding of wild pigs mainly concentrated in the Prairie Provinces with some of the coldest winters of all ten provinces and that the species is rare or absent in the warm coastal areas is inconsistent with studies that have found that wild pig distribution is positively associated with warmer climates and have suggested cold winter temperatures to be highly limiting^10,37^. This likely reflects, in part, that domestic wild boar farms that are known sources of free-ranging wild pigs were, and are, more concentrated on the Canadian Prairies^19^. The success of wild pigs in western Canada does highlight the capacity for wild pigs to thrive and expand in areas with long and extremely cold winters including some occurrences north of 55° north latitude.

The majority of occupied watersheds throughout Canada are located in agriculture and human-dominated landscapes. The presence of wild pigs in these landscapes creates significant socio-economic risks incurred from economic loss from crop damage^43^, predation of small and young livestock^9,22^, vehicle collisions^44^, structural damage^26^, and health and safety concerns to humans, livestock, and wildlife^16,45^. Although these risks have yet to be quantified in Canada, agriculture losses in the United States from wild pig damage have been estimated at $1.5 billion USD per year by Pimental^46^, based on extrapolation of localized data. Wild pig-vehicle collisions cost $36 million USD per year^44^, and there is the continued threat of significant economic losses that could be incurred to the livestock industry and international trade if diseases reportable to the Canadian Food and Inspection Agency were identified^45,47^. In many areas of their introduced and native range wild pigs are considered disease reservoirs and maintenance hosts due to their high densities, complex social behaviors, and ability to maintain the disease without a continued source of infection^48–50^. Wild pigs are host to 89 bacterial, viral, and parasitic diseases which can be transferred to livestock, wildlife, and humans^16,45,51^. Common diseases of concern transmitted from wild pigs to livestock are swine brucellosis, bovine tuberculosis, pseudorabies, and classical swine fever, while zoonotic diseases include brucellosis, *Escherichia coli*, salmonellosis, and leptospirosis^16,45,52,53^. Diseases at the livestock-wildlife interface pose challenges to wildlife managers and livestock producers, as well as towards quantitative risk analyses, as the disease status of wildlife populations and routes of transmission are often poorly understood^45,51^. Disease threats are an increasing concern to livestock producers and the pork and beef industries, as disease outbreaks are associated with high economic losses^9,51,54^. The challenges associated with eliminating disease in wildlife populations pose a continued threat of disease introduction to livestock, act as an impediment to disease elimination in livestock populations, and has the potential for spill-over back into wildlife populations^49,55,56^.

While our mapping efforts provide national scale coverage for an area of 5.5 million km^2^ at a relatively low cost and the benefits of this approach are evident, there are also limitations that should be considered. Overall, correspondence of wild pig detection between methods was low, with none of the methods came close to detecting all of the occupied watersheds identified by all methods combined. This lack of saturation demonstrates the value of an approach based on multiple methods for identifying a large dataset of novel detections, but also highlights that while stakeholder snowball sampling detected the most wild pig observations within watersheds, they only identify less than half of all watersheds detected in this study. No single method provided comprehensive coverage of the study area due to different sampling designs, stratification units, and response rates of different target groups. The arbitrary stratification units used to determine response for the different sampling methods provided different sizes of coverage between and within methods, with a general trend in unit size increasing northward, and decreasing in size towards the south. The combined use of multiple methods provided comprehensive coverage of the study area. Although it is expected that non-detection errors occurred, the extensive coverage of the study area provides confidence in the overall distribution of wild pigs across Canada.

In presence-absence survey methods non-detection errors and false absences are a common sampling problem^57,58^. Due to the elusive and often nocturnal behavior of wild pigs, their preferred habitat comprised of thick cover in wetlands and forests, and relatively low density across most of the study area, some false negatives are expected. Using interviews with large numbers of study participants and multiple independent methods helped address challenges in detecting wild pigs and balancing the benefits and limitations of each method^59,60^. We recognize that our combined map of watersheds identified with wild pigs represents a minimum estimate and that there are likely some undetected watersheds, but given such rapid expansion of wild pigs this is inevitable. Non-detection was addressed in the survey design and implementation with the use of multiple survey methods across the study area and a large sample size^59,60^. The possibility of false positives by misidentifying species is also a concern in presence/absence surveys that has the potential to overestimate presence^60^, however, wild pigs are a novel, large, and distinctively shaped species on the landscape that are unlikely to be misidentified. Additionally, the majority of our study participants are individuals who live, work, and pursue recreational activities outdoors, therefore have a higher than average exposure to, and knowledge of, wildlife.

The overall goal of our comparison of different methods was to promote consideration of a wide range of options and how to evaluate and integrate them, not to be prescriptive. Each study will have different budgets, time constraints, and access to existing datasets and so will make different decisions. Expert interviews through systematic sampling provided the most widespread range of independent, unique locations across the study area at moderate cost. The use of expert elicitation is a common method used in identifying species distributions and is a valuable resource^61,62^, however is potentially limited by non-response bias. Non-response within the expert elicitation survey was found to be affected by the distribution of, and individual’s knowledge regarding, wild pigs; however the most significant factor in determining response rate was the time constraints of experts due to priorities within their profession. Therefore, non-response, while in some cases was determined by the lack of wild pig presence, did not follow the distribution of wild pigs and is not considered to have biased the expert interview method.

Access to social networks of stakeholders using snowball sampling provided the largest number of occupied watersheds across a broad spatial scale at a relatively low cost. The use of social networks allows for large sample sizes to be obtained at a low-cost from hard-to-reach populations with unknown parameters^63^. However, this technique can introduce selection bias since it involves non-probability sampling of a specific target group, where over or under-representation of a group or specific characteristic can occur due to stronger or weaker social connections^64^. Inherently, selection bias was present in our sampling design, as only individuals with wild pig knowledge and within subsequent referral chains participated. However, we addressed this in the sampling design through the large sample of participants, as well as by access to several small, discrete referral chains, rather than a few large ones^65,66^ therefore were able to access a range of participants across a broad geographic and temporal scale.

The rural telephone survey was only moderately effective in terms of the number and range of wild pig detections compared to the high cost. However, the random stratified sampling method and large sample size across all ten Canadian provinces provided some novel data and had limited bias associated with the sampling technique. The stratified random sampling technique reduced bias, as individuals were randomly selected and a set number of interviews was previously identified, therefore non-response bias in provinces without, or with very few, wild pigs was limited by the survey design. The contribution of this sampling method to the overall study design was that it helped examine whether there was any bias in data obtained from the other social science sampling methods. While the overall number of detected watersheds was low for the rural telephone survey, the distribution of occupied watersheds followed the same trend observed in the other social survey methods, validating that bias had been reduced in the expert interview and stakeholder snowball sampling methods. The bounty program in the province of Alberta provided a large number of wild pig locations relative to its small extent, but was limited to specific counties within a single province. While bounties are considered counter-productive to wild pig control effort^67^  they did help identify the provincial distribution in Alberta.

Methods used in the evaluation and validation of the sampling methods and wild pig locations included GPS collars, citizen science and research trail camera network photos, and media searches. These methods were not suitable for including in the national-scale mapping, but were valuable in evaluating the costs, limitations, and benefits associated with different sampling methods and validating wild pig detections. The use of GPS collars is a common technique in wildlife research, providing multiple locations daily with high spatial accuracy^68^. However, this was by far the most expensive method evaluated, and while it provided a large number of occurrence points it only occurred at a fine scale, with few watersheds detected overall. Similar research evaluating multiple sampling methods has demonstrated the value of GPS collars and radio telemetry for evaluation or validation of multiple sampling methods, as opposed to an improvement or ‘truth’ in distribution mapping or occupancy estimation^31,32^. Research trail camera networks and citizen science photos were a low-cost, spatially accurate, and unbiased means of validating wild pig locations. Media searches resulted in very few, broad-scale detections and were difficult to validate through additional contact with the primary information holder.

The combined use of multiple methods that incorporates social and ecological data has been documented in the literature and is an effective tool for large-scale data collection of a species^31–33,69–71^. Large sample sizes and the incorporation of multiple sampling methods and survey designs conducted in this study provided a more robust dataset, captured a wider range of information holders, and reduced sampling bias^69,70,72^. As such, we did not choose one method as ‘truth’ against which to compare all others, but rather to consider all methods as any other dataset to evaluate benefits and limitations and compare with the database of all combined occurrences^73^. Several studies have found that a strong correlation exists between traditional or local ecological knowledge and western science, with the differences between the two ultimately stemming from differences in temporal and spatial scale^31,32,74,75^. When used in conjunction with one another, the variation in spatial and temporal scales between local and traditional knowledge with conventional ecological research provides complimentary and novel information and can address gaps in knowledge^31,76,77^.

Timely and cost-effective mapping of invasive species over large areas (>1 million km^2^) is essential to developing effective management strategies and responding rapidly to introduced species and rapidly changing distributions. Our study design utilized multiple sampling methods to acquire a large dataset, compare and evaluate results, and identify the national distribution and range expansion of a large, low-density, nocturnal species. The overall approach that we present here can be applied to species distribution studies, with a strong emphasis on invasive species. Although study designs may vary according to the ecology and behavior of the species being studied and the timing of introduction events, the transdisciplinary approach we developed and validated effectively addresses the common challenges and provides a process that is broadly relevant to any invasive species. Each method evaluated has benefits and limitations associated with it, and trade-offs between methods need to be assessed for individual studies. The rapid identification of invasive species distribution provides managers with the ability to implement management strategies to attempt to eradicate the species before population and density levels increase to the point where the cost of eradication becomes unfeasible.

Our map provides the first documented distribution of invasive wild pigs across Canada’s ten provinces. The widespread distribution and significant range expansion identified over the past 27 years, along with the extensive and costly list of risks associated with wild pigs, highlights the need for rapid and aggressive management action. The map offers a baseline against which future range expansion can be compared and a valuable resource for identifying priority management and conservation areas. In the current millennium with ever expanding globalization, land use changes, and climate change, challenges associated with invasive species are likely to only increase^5,78^. Future research can apply the technique established in this study for rapid and cost-effective identification and understanding of the distribution of an invasive species on the landscape.

## Methods

### Study area

Our study area included all ten Canadian provinces. Bordered by the Pacific Ocean to the west and the Atlantic Ocean to the east, Canada’s provincial landmass has an area of 5,499,918 km^2^ ^79^ and stretches from the U.S./Canada border northward to 60°00′ N latitude. This large expanse of land includes a broad range of biodiversity and variability in ecosystems, topography, temperature, and precipitation that includes mountain ranges, open grassland, temperate rainforest, coniferous and deciduous forest, plains, and boreal shield^80^. As a result of the heterogeneity across latitude, elevation, and proximity to the moderating effects of large waterbodies, or the more extreme inland climates, considerable variability in temperature and precipitation is observed across Canada’s 15 ecozones^81,82^.

Our preliminary research based on interviews with researchers and wildlife managers across Northern Canada’s three territories (Yukon, Northwest Territories, and Nunavut) who further consulted with field staff and stakeholders, determined that there was no evidence of wild pigs in these most northerly ecozones, as predicted based on the literature, the extreme cold winter temperatures (<−50 °C) combined with long winters (>7 months), and the absence of past or present domestic wild boar production (except several recent small operations near Whitehorse, Yukon) (Brook unpublished data) which are the source of invasive wild pigs. As such we excluded Taiga Cordillera, Taiga Plans, Taiga Shield, Hudson Plains, Southern Arctic, Northern Arctic, Arctic Cordillera, and Tundra Cordillera ecozones and defined our current study area as including the Pacific Maritime, Montane Cordillera, Boreal Plains, Boreal Shield, Prairies, Mixedwood Plains, and Atlantic Maritime ecozones.

### Data collection comparisons

We collected four independent wild pig occurrence datasets and four evaluation/validation datasets using social science and conventional wildlife monitoring to document and evaluate wild pig occurrences across Canada. Our social survey design was approved by the University of Saskatchewan Behavioral Research Ethics Board (BEH# 15–155) and the ecological data was approved by University of Saskatchewan Animal Research Ethics Board (Animal Use Protocol no. 20150024). Both human and wildlife research performed in this study were in accordance with the terms of the ethics approvals above. Informed consent was obtained for all human participants throughout all of the described data collection methods. While the term ‘wild pig’ is now widely used^6^, for our survey we used the term ‘wild boar’ as we found from preliminary consultations and pre-testing that this term was best understood at the time of the study by rural Canadians. We are now consistently using ‘wild pigs’, recognizing that few, if any, pure bred domestic or free-ranging Eurasian wild boar exist in Canada. The datasets were evaluated against one another to assess the trade-offs that occur from different data collection methods. The total number of wild pig detections and number of occupied watersheds were calculated along with the total cost and cost per wild pig detection for each data collection method.

### Expert interview survey design

A stratified sampling design based on provincial wildlife enforcement and management districts was conducted from January 2015 to December 2017. At least one conservation officer (C.O.) and one government wildlife biologist in each district was contacted where possible. Contact information and locations were found through employee directories on provincial government websites. The first positive response received was the individual with which the interview was completed. Three contact attempts were made for each individual, with a message left on the first attempt. Attempts were halted once one interview within the strata had been completed or there was no response after three attempts. If no interviews were completed within a stratum it was considered a non- response. Strata were considered half-complete if both a C.O. and biologist were located in the stratum, but only one of the two completed an interview. Interview questions are included in Supplementary Methods (S1).

### Stakeholder snowball sampling design

A snowball sampling survey design^83^ was implemented to access key participants with knowledge of wild pig presence. Sampling took place from October 2014 to December 2017. Recruitment for this study began with an individual who shared their observations and then provided additional contacts that were also likely to know of wild pig occurrences. This snowball method of accessing additional contacts creates an ever-expanding network of individuals with information^64,71^. The technique uses social networks; therefore, personal communication between the researchers and members of the social networks was essential^64^. Non-probability sampling methods typically require the use of advertising and outreach to be successful, compared to the stricter framework of probability sampling, in which letters and phone calls are made to specific individuals^84^. The snowball sampling method for this study used both techniques for recruiting participants. Multiple methods of recruitment were utilized to gain access to participants. Presentations at wildlife meetings and conventions, information booths, posters, and magazine articles were all forms that were used to recruit participants. Active recruitment such as presentations and information booths were used to make initial contacts and gain access to social networks. Face-to-face contact was primarily used for initial contact and telephone was used to access additional contacts. Passive recruitment in the form of posters and magazine articles were used in an attempt to access an even broader geographic range of participants. Individuals with first-hand wild pig knowledge were also provided by the sample of experts. In order to keep the interest of the participant, and hence response rate, high, interviews were not completed with snowball sample members, only questions relating to wild pig locations were asked.

### Rural telephone survey design

We used a stratified random sampling design to conduct a telephone survey of 3,000 individuals, representative of the rural Canadian population. The survey instrument included several themes but for the purposes of this paper we only used the responses to three questions: (1) What is your postal code? (2) Are you aware of wild boar presence or observations in your area in the past five years? (3) If yes, can you provide a date and location of the observation? Data collection was conducted by the Social Science Research Laboratory call center at the University of Saskatchewan. Participants could respond in either official language, English or French, and French responses were translated by individuals with a strong proficiency in that language. Provinces were stratified based on Forward Sortation Area’s (FSA’s) for rural areas as defined by Canada Post. The interview quota for each province was stratified based on the provincial rural population (from Stats Canada 2017 unpublished data) and individual contact information was randomly selected from within the FSA’s. Interviewers asked to speak with a member of the household that was over the age of 18 and was having the next birthday. Interviews were completed in February and March of 2016 to take advantage of the time when farmers were least busy. Individuals with wild pig information were asked if they would be willing to provide their contact information and receive a follow-up call from the primary researcher. Follow-up calls inquired about wild pig locations in greater detail as a means of validation. Interview questions and scripts are included in Supplementary Methods (S1).

### Bounty data collection

A bounty on wild pigs was in effect in the province of Alberta from 2003 to 2016. The program was initiated by the provincial government with individual counties signing up to participate. Hunters received $50 for every pair of wild pig ears that were turned into the county, and were required to provide the associated location and date of the kill. All counties participating in the program were contacted and data regarding the location, date, and number of pairs of ears turned in was received. Data were then compiled and verified.

### GPS collars

We captured 21 female and 17 male wild pigs by net-gun fired from a helicopter (n = 33) and through trapping using corral-style traps (n = 5) from 2015 to 2017 in southeastern and east-central Saskatchewan. After capture, each wild pig was physically immobilized and fitted with a Global Positioning System (GPS) tracking collar (Telonics, Mesa Arizona, USA). All collars were programmed to record a location every three hours and transmit the data via Iridium satellite link.

### Citizen science photos

Citizen science photos consisted of images from trail cameras, digital cameras, and cellular phones. Photos were provided by stakeholders and experts as supplementary information associated with wild pig locations when available.

### Trail camera network

We used images from a previous study (O’Brien *et al*., unpublished data) in our study design as an evaluation/validation method. Over a two-year period from 2011 to 2013 a network of 17 research trail cameras were deployed to capture wild pigs over an area of 275 km^2^ in east-central Saskatchewan (O’Brien *et al*., unpublished data).

### Media search

Media searches were conducted between 2014 and 2017. Searches were conducted in Google, media outlets, hunting forums, and social media and included key words such as “Eurasian wild boar”, “wild boar”, “feral boar”, “feral wild boar”, “feral pig”, and “wild pig”.

### Response coverage

Coverage by each method was determined using pre-defined provincial and national units that provided stratified and comparable coverage across the entire study area. Experts were stratified by provincial wildlife management zones, which are designated areas they manage wildlife and work within. The snowball stakeholder and bounty methods used provincial municipal administrative units and the telephone survey used national Forward Sortation Areas. All methods combined used the Canadian census sub-division units, as this stratification unit was consistent across the entire study area and provided a conservative unit size at the national scale with an average area of 1,195 km^2^ ^85^.

### Wild pig occurrence mapping

We followed the MaNIS Georeferencing protocol (2001) and converted all wild pig locations into UTM coordinates using Google Maps (2018) and the Legal Land Description Converter (2017). Estimates of spatial accuracy of the data were based on information from the provider and using map scale. Two Level 9 (North America and Arctic) watershed shapefiles were downloaded from HydroSHEDS (2017) following the Pfafstetter coding system^86^. The 37,578 Level 9 watershed sub-basins in the study area have a mean area of 267 km^2^ ^87^. Watershed units were chosen to model wild pig distribution as they are ecologically stratified units and allow for consistency across the landscape based on abiotic and biotic factors^10,32,88^. Level 9 watershed sub-basins are large enough to include the home range of at least one individual or sounder, which average 3.6 km^2^ for sows and 4.91 km^2^ for boar^89–92^, while encompassing considerable landscape heterogeneity^10^, The use of watershed units also maintains confidentiality of exact locations to ensure private land is not easily accessible to hunters, which was a condition of many landowners for providing information. Each wild pig occurrence was buffered by a radius of 10 km, a conservative estimate of annual home range movements and an area larger than the expected spatial error from any of the data collection methods. Watersheds that intersected a wild pig location were selected to create a watershed occurrence layer illustrating wild pig presence by watershed units. Two ten-year interval maps were created from 1990–2000 and 2001–2010. A current wild pig location map was created for the years 2011–2017.

## Supplementary information

Interview Questionnaires

## Data Availability

Aggregate data are provided in this paper. Fine-scale point locations are required to remain confidential in accordance with our ethics approval (U of S BEH# 15–155).
