# Supplementary material for: Evaluating Cost-Effective Methods for Rapid and Repeatable National Scale Detection and Mapping of Invasive Species Spread
Source: Sci Rep. 2019 May 10;9:7254. doi: 10.1038/s41598-019-43729-y (PMC6510748; doi:10.1038/s41598-019-43729-y)
Supplement: Supplementary file 1 — Interview Questionnaires [file 41598_2019_43729_MOESM1_ESM.pdf]

Evaluating Cost-Effective Methods for Rapid and Repeatable National Scale Detection and Mapping of Invasive Species Spread

Ruth A. Aschim and Ryan K. Brook

University of Saskatchewan
College of Agriculture and Bioresources

Feral Boar Observations and Perceptions Questionnaire for Target Group of Experts

Date:____

Surveyor:_______________________________________

Interview #:_____________________________________

Name:____

Province:______________________________________

Geographic Location:______________________________

Occupation:_____________________________________

Map Attached:___________________________________

Photos:_______________________________________

Contact Info:____________________________________

Additional Notes/Contacts:__________________________

____

____

____

Part A.

1. Are you aware of feral boar presence in your province in the past two years?

Yes	No	I Don't Know

2. If yes, what area(s) of the province are feral boar present?

____

3. Are you aware of feral boar presence or observations in your jurisdiction in the past two years?

Yes	No	I Don't Know

4. If yes, how often does feral boar sign or presence occur in your jurisdiction?

Very Rarely	Rarely	Sometimes	Commonly	Often	Always	I Don't Know

5. How long has it been since you have been aware of feral boar in your area?

Days	Weeks	Months	Years	Never

- Are you able to provide a more detailed time-frame?

____

6. Are you aware of feral boar sign in your area?

Tracks	Scat	Rooting	Wallowing	Rubs	Crop Damage	Livestock Damage	Structural Damage	I Don't Know

7. In what habitats have feral boar or feral boar sign been observed?

Cropland	Perennial Forage	Pasture	Grassland	Forest	Shrub Land
Wetlands	Rivers/Lakes	Valleys/Hills	Roads	Other	I Don't Know

- If other, please describe

____

8. At what time of day are feral boar most commonly observed?

Dawn	Morning	Afternoon	Dusk	Night	I Don't Know

9. What months are feral boar or feral boar sign most commonly observed?

January	February	March	April	May	June	July
August	September	October	November	December	All Year	I Don't Know

10. If feral boar have been observed, can you estimate the relative abundance observed over the past two years?

None	Very Few	A Few	Abundant	I Don't Know

11. Are feral boar commonly observed in:

Groups (sounders)	Mother With Piglets	Solitary	I Don't Know

12. Are you aware of any currently active domestic wild boar farms in your area?

Yes	No	I Don't Know

If yes,

- How many? ________________________
- Are you aware of any animals escaping to the wild from domestic boar farms?

Yes	No	I Don't Know

13. Are you aware of any domestic wild boar farms in your area in the past 20 years that used to exist, but are no longer in operation?

Yes	No	I Don't Know

If yes,

- How many? ________________________
- Are you aware of any animals escaping to the wild from domestic boar farms?

Yes	No	I Don't Know

14. Are you aware of feral boar hunting in your area?

Yes	No	I Don't Know

If yes,

- How would you describe hunting pressure in your area?

None	Low	Moderate	Heavy	I Don't Know

- Does hunting occur throughout the year?

Yes	No	I Don't Know

- If no, when time(s) of the year does hunting occur?

  ____

For the following questions please answer in terms of your agreement to the question (1=strongly disagree, 5= neutral, 10=strongly agree) or I Don't Know

15. Do you perceive feral boar presence in Canada as a critical current concern?
16. Do you perceive feral boar presence in your province as a critical current concern?

17. Do you perceive feral boar presence to become a large problem in Canada in the future?
18. Do you perceive feral boar presence to become a large problem in your province in the future?
19. Do you feel greater feral boar eradication and control action in Canada is required?
20. Do you feel greater feral boar eradication and control action in your province is required?
21. Do you think feral boar creates exciting new hunting properties in Canada?
22. Do you think feral boar creates exciting new hunting opportunities in your province?

23. What population trend would you like to see regarding feral boar in your area?

No Feral Boar	A Decrease	Remain Stable	An Increase	I Don't Know

24. Are you aware of any current or past feral boar management or control programs in your province?

Yes	No	I Don't Know

If yes,

- What are they?

  ____

- Have they been effective in your jurisdiction?

  ____

For the following questions please answer from 1 to 10 (1=none, 5=neutral, 10=high) or I Don't Know

25. How would you rate current public knowledge of feral boar in Canada?
26. How would you rate current public knowledge of feral boar in your province?
27. How would you rate the level of pressure from stakeholders, wildlife managers, and the public towards government for greater feral boar management and control strategies?

28. How serious has damage caused by feral boar been in your province in the last two years?

Never Serious	Seldom Serious	Serious Sometimes	Serious All The Time	I Don't Know

29. How serious has damage caused by feral boar been in your area in the last two years?

Never Serious	Seldom Serious	Serious Sometimes	Serious All The Time	I Don't Know

Please indicate your level of concern for each issue regarding feral boar
(1=no concern, 5=moderate concern, 10=extremely high concern) or I Don't Know

30. Crop damage
31. Livestock predation and harassment
32. Structural damage
33. Environmental impacts
34. Threat to native wildlife
35. Disease transmission to wildlife and livestock
36. Disease transmission to humans
37. Threats to human safety and well-being
38. Other

<u>Part B.</u>

1. What area does your jurisdiction cover? (RM's, counties, park boundaries, etc.)
____

2. How long you have you worked in this profession?
____

3. How long have you held your current position?
____

4. If feral boar are present within your jurisdiction, when were they first observed/introduced?
____

5. How often do you receive public inquiries regarding feral boar sightings, feral boar hunting laws and regulations, feral boar hunting locations, feral boar damage, and general feral boar questions?

Never	Rare	Sometimes	Common	Abundant	I Don't Know

6. Which, if any, inquiries are more common than others?
____

7. From what stakeholder group do you receive the most inquiries about feral boar?
____

8. How do you perceive feral boar population trends in your jurisdiction over the past 5 years?

Greatly Decreased	Somewhat Decreased	Remained Stable	Somewhat Increased	Greatly Increased	I Don't Know

9. What factors do you think have led to this population trend in your jurisdiction?
____

Rural Telephone Survey Script for use by the University of Saskatchewan Social Science Research Laboratory

INTRO1./INTRO3.

Hello, my name is ___***(FIRST NAME ONLY)***___ and I am calling on behalf of Dr. Ryan Brook and Ruth Kost at the University of Saskatchewan. We are conducting a short telephone survey as part of a national study on wild boars in rural Canada.

Bonjour, mon nom est ___***(FIRST NAME ONLY)***___ et je vous appelle au nom de M. Ryan Brook et Ruth Kost à l'Université de la Saskatchewan. Nous menons une coute enquête téléphonique dans le cadre d'une étude nationale sur les sangliers sauvages dans les régions rurales du Canada.

WILD BOAR DEFINITION – HARD COPY ONLY:

Eurasian Wild Boar that are outside of a fence. Can be outside of a fence for generations or recently. Large animal with dark brown/black hair, males have tusks. All Canadian wild boar originated from domestic wild boar farms and have either escaped or been released from farms.

Les sangliers sauvages d'Eurasie qui sont à l'extérieur d'une clôture. Ils peuvent être à l'extérieur d'une clôture pendent les générations ou récemment. Ils sont des grands animaux avec des poils noirs/bruns foncés, les mâles ont des défenses. Tous les sangliers canadiens sont provenus des fermes de sanglier domestiques et ils ont échappés ou a été libérés des fermes.

INTRO2.

May I please speak with the person in your household who is 18 years of age or older and having the next birthday?

Est-ce que je peux parler avec la personne de votre ménage qui est agé de 18 ans ou plus et ayant le prochain anniversaire?

1. Yes, speaking ***CONTINUE***
2. Yes, I'll get him/her ***REPEAT INTRODUCTION AND CONTINUE***
3. Not available ***ARRANGE CALLBACK - REQUEST RESPONDENT FIRST NAME***

(RECORD IN NOTES) AND ARRANGE CALLBACK (PRESS THE CTRL AND END KEYS)

INTRO4.

I would now like to invite you to participate in a short (***5-10-minute***) survey. Participation is voluntary, and you can stop the survey at any time. Let me assure you that the information we collect is kept strictly confidential and none of the answers that you provide will be attributed to you personally. If you would like more information on the study, please contact Ruth Kost at 306-227-0998. If you have any concerns or questions about the survey, you may contact the Research Ethics Office at the University of Saskatchewan toll free at 1-888-966-2975.

Are you willing to participate in the survey?

Je voudrais maintenant vous inviter à participer à une enquête courte de 10 minutes. La participation est volontaire, et vous pouvez arrêter l'enquête à n'importe quel moment. Permettons-nous de vous assurer que les informations que nous recueillons sont strictement confidentielles et aucune des réponses que vous fournissez sera attribué à vous personnellement. Si vous souhaitez plus d'informations sur l'étude, s'il vous plaît contacter Ruth Kost au 306-227-0998. Si vous avez des problèmes ou des questions concernant l'enquête, vous pouvez communiquer avec le bureau d'éthique de la recherche à l'Université de la Saskatchewan sans frais au 1-888-966-2975.

Voulez-vous participer à l'enquête?

1. Yes ***CONTINUE***

2. No ***THANK AND END INTERVIEW***

3. Later/Not right now ***ARRANGE CALLBACK - REQUEST RESPONDENT FIRST NAME***

(RECORD IN NOTES) AND ARRANGE CALLBACK (PRESS THE CTRL AND END KEYS)

INTRO5.

Before we begin, can I please have your postal code?

Avant de commencer, est-ce que je peux avoir votre code postal s'il vous plaît?

IF RESPONDENT IS RELUCTANT, YOU CAN ASSURE THEM THAT THEIR POSTAL CODE WILL BE USED FOR STATISTICAL PURPOSES ONLY (TO UNDERSTAND DIFFERENCES BY REGION/GEOGRAPHY) AND WILL NOT BE USED TO IDENTIFY THEM IN ANY WAY.

ENSURE RESPONDENT PROVIDES COMPLETE SIX CHARACTER POSTAL CODE IN PROPER FORMAT (EXAMPLE: S7N 5A5)

ENTER RESPONDENTS POSTAL CODE:

1. (RECORD POSTAL CODE)

INTRO6.

(DO NOT READ)

RECORD SEX FROM RESPONDENT VOICE.

1. Male
2. Female

A1.

Are you aware of wild boar presence or observations in your area in the past five years?

Etes-vous conscient de la présence ou des observations des sangliers sauvages dans votre région au cours des dernières cinq années?

(IF RESPONDENT IS RELUCTANT, PLEASE ASSURE THAT LOCATIONS ARE CONFIDENTIAL AND USED FOR RESEARCH PURPOSES ONLY)

(READ LIST IF NECESSARY)

1. Yes
2. No ***(IF SELECTED, SKIP TO A3)***
9. (Don't Know) ***(IF SELECTED, SKIP TO A3)***
10. (Refused) ***(IF SELECTED, SKIP TO A3)***

A2.

(IF YES TO A1)

I would like to ask you some questions about your observation of wild boar presence in your area in the past five years.

What was the location and year of the sighting(s)?

Je voudrais vous poser quelques questions au sujet de vos observations de la présence des sangliers sauvages dans votre région au cours des dernières cinq années.

Quel était l'emplacement et l'année de l'observation(s)?

(IF MORE THAN 3 SIGHTINGS ARE NOTED, RECORD THE 3 MOST RECENT SIGHTINGS)

(LOCATION OPTIONS COULD INCLUDE LAND LOCATION, UTM COORDINATES, OR DISTANCE AND DIRECTION TO THE NEAREST TOWN – ANY APPROXIMATE LOCATIONS ARE OKAY)

(READ LIST IF NECESSARY)

Sighting 1:

1. The location Please specify)
2. The year (Please specify)
9. (Don't Know)
10. (Refused)

Sighting 2:

1. The location (Please specify)
2. The year (Please specify)
9. (Don't Know)
10. (Refused)

Sighting 3:

1. The location (Please specify)
2. The year (Please specify)
9. (Don't Know)
10. (Refused)

A3.

Is wild boar presence on your property a concern to you? Please indicate whether it is of no concern, low concern, neutral concern, moderate concern, or high concern.

Est-ce que la présence de sangliers sauvages sur votre propriété une inquiétude pour vous? S'il vous plaît indiquer si ce n’est pas une inquiétude, si c’est une inquiétude mineure, si c’est une inquiétude neutre, si c’est une inquiétude modérée ou une inquiétude élevée.

(READ LIST IF NECESSARY)

1. No concern
2. Low concern
3. Neutral concern
4. Moderate concern
5. High concern
6. (Don’t Know)
7. (Refused)

B1.

For the following statements please use a scale from 1 to 10, where 1= strongly disagree, 5= neutral, and 10= strongly agree.

Pour les énoncés suivants s'il vous plaît utiliser une échelle d’un à 10, où 1 est égal à fortement en désaccord, 5 est égal à neutre, et 10 est égal à fortement en accord.

(RANDOMIZE QUESTIONS B1a to B1e)

B1a.

Wild boar presence in your province is a critical current concern.

La présence des sangliers sauvages dans votre province est une inquiétude cruciale actuelle.

(READ LIST IF NECESSARY)

1. 1 = Strongly disagree
2. 2
3. 3
4. 4
5. 5 = Neutral
6. 6
7. 7
8. 8
9. 9
10. 10 = Strongly agree
11. (Don’t Know)
12. (Refused)

B1b.

Wild boar presence will become a large problem in your province in the future.

La présence des sangliers sauvages va devenir un grand problème dans votre province à l'avenir.

(READ LIST IF NECESSARY)

1. 1 = Strongly disagree
2. 2
3. 3
4. 4
5. 5 = Neutral
6. 6
7. 7
8. 8
9. 9
10. 10 = Strongly agree
11. (Don’t Know)
12. (Refused)

B1c.

Wild boar management in your province is required.

La gestion des sangliers sauvages dans votre province est nécessaire.

(READ LIST IF NECESSARY)

1. 1 = Strongly disagree
2. 2
3. 3
4. 4
5. 5 = Neutral
6. 6
7. 7
8. 8
9. 9
10. 10 = Strongly agree
11. (Don’t Know)
12. (Refused)

B1d.

Wild boar should be eradicated from your province.

Les sangliers sauvages doivent être éradiqués de votre province.

(READ LIST IF NECESSARY)

1. 1 = Strongly disagree
2. 2
3. 3
4. 4
5. 5 = Neutral
6. 6
7. 7
8. 8

9. 9
10. 10 = Strongly agree
11. (Don't Know)
12. (Refused)

B1e.

Wild boar creates exciting new hunting opportunities in your province.

Les sangliers sauvages créent des nouvelles opportunités de chasse dans votre province.

(READ LIST IF NECESSARY)

1. 1 = Strongly disagree
2. 2
3. 3
4. 4
5. 5 = Neutral
6. 6
7. 7
8. 8
9. 9
10. 10 = Strongly agree
11. (Don't Know)
12. (Refused)

C1.

For our final section of statements, please indicate your level of concern for the following issues regarding wild boar. Please use a scale from 1 to 10, where 1= no concern, 5= moderate concern, and 10= extremely high concern.

Pour notre dernière section d'énoncés, s'il vous plaît indiquer votre niveau de préoccupation pour les questions suivantes concernant les sangliers sauvages. S'il vous plaît utiliser une échelle d'un à 10, où 1 est égal à pas d' inquiétude, 5 est égal à une inquiétude modérée, et 10 est égal à une inquiétude extrêmement élevée.

(RANDOMIZE QUESTIONS C1a to C1i)

C1a.

Crop damage.

Les dommages aux recoltes.

(READ LIST IF NECESSARY)

1. 1 = No concern
2. 2
3. 3
4. 4
5. 5 = Moderate concern
6. 6
7. 7
8. 8
9. 9
10. 10 = Extremely high concern
11. (Don’t Know)
12. (Refused)

C1b.

Livestock predation and harassment.

La prédation et le harcèlement du bétail

(READ LIST IF NECESSARY)

1. 1 = No concern
2. 2
3. 3
4. 4

5. 5 = Moderate concern
6. 6
7. 7
8. 8
9. 9
10. 10 = Extremely high concern
11. (Don’t Know)
12. (Refused)

C1c.

Structural damage.

Les dommages structurels.

(READ LIST IF NECESSARY)

1. 1 = No concern
2. 2
3. 3
4. 4
5. 5 = Moderate concern
6. 6
7. 7
8. 8
9. 9
10. 10 = Extremely high concern
11. (Don’t Know)
12. (Refused)

C1d.

Environmental impacts.

Les impacts sur l’environement.

(READ LIST IF NECESSARY)

1. 1 = No concern
2. 2
3. 3
4. 4
5. 5 = Moderate concern
6. 6
7. 7
8. 8
9. 9
10. 10 = Extremely high concern
11. (Don’t Know)
12. (Refused)

C1e.

Threat to native wildlife.

La menace pour les animaux indigènes.

(READ LIST IF NECESSARY)

1. 1 = No concern
2. 2
3. 3
4. 4
5. 5 = Moderate concern
6. 6
7. 7
8. 8
9. 9
10. 10 = Extremely high concern
11. (Don’t Know)
12. (Refused)

C1f.

Disease transmission to wildlife and livestock.

La transmission des maladies aux animaux indigènes et aux bétails.

(READ LIST IF NECESSARY)

1. 1 = No concern
2. 2
3. 3
4. 4
5. 5 = Moderate concern
6. 6
7. 7
8. 8
9. 9
10. 10 = Extremely high concern
11. (Don't Know)
12. (Refused)

C1g.

Disease transmission to humans.

La transmission des maladies aux êtres humains.

(READ LIST IF NECESSARY)

1. 1 = No concern
2. 2
3. 3
4. 4
5. 5 = Moderate concern
6. 6
7. 7
8. 8
9. 9
10. 10 = Extremely high concern
11. (Don't Know)
12. (Refused)

C1h.

Threats to human safety and well-being.

La menaces à la sécurité des êtres humains et leur bien-être.

(READ LIST IF NECESSARY)

1. 1 = No concern
2. 2
3. 3
4. 4
5. 5 = Moderate concern
6. 6
7. 7
8. 8
9. 9
10. 10 = Extremely high concern
11. (Don't Know)
12. (Refused)

D1.

(IF YES TO A1)

Lastly, I am wondering if you would be willing to be contacted by the primary researcher on this study regarding your knowledge of wild boar?

Enfin, je vous demande si vous êtes disposé d'être contacté par le chercheur principal de cette étude au sujet de votre connaissance des sangliers sauvages?

1. Yes
2. No

D1a.

(IF YES TO D1)

(IF RESPONDENT IS RELUCTANT, THEY CAN BE ASSURED THAT THEIR CONTACT INFORMATION WILL NOT BE SHARED AND THEIR RESPONSES PROVIDED IN THE SURVEY WILL REMAIN CONFIDENTIAL)

Please provide your first name and a telephone number the primary researcher could contact you at:

S'il vous plaît pourriez-vous fournir votre prénom et un numéro de téléphone pour que le chercheur principal puisse vous contacter:

1. First Name:
2. Telephone:
9. (Refused)

E1.

Those are all the questions that I have! For more information on the study itself, please contact Ruth Kost. On behalf of the University of Saskatchewan, thank you for your time. Your responses are greatly appreciated! Have a great day/evening!

Ce sont toutes les questions que j'en ai! Pour plus d'informations sur cette étude, s'il vous plaît contactez Ruth Kost. Au nom de l'Université de la Saskatchewan, je vous remercie pour votre temps. Vos réponses sont très appréciées! Passez une bonne journée / soirée!
